# Posttraumatic arthritis and functional outcomes of nonoperatively treated distal radius fractures after 3 years

**DOI:** 10.1038/s41598-023-48630-3

**Published:** 2023-11-30

**Authors:** Rikke Thorninger, Karen Larsen Romme, Daniel Wæver, Martin Bille Henriksen, Michael Tjørnild, Martin Lind, Jan Duedal Rölfing

**Affiliations:** 1 Department of Orthopaedics, Regional Hospital Randers, Skovlyvej 15, 8930 Randers, Denmark; 2 Department of Clinical Medicine, HEALTH, Aarhus University, Palle Juul-Jensens Boulevard 82, 8200 Aarhus, Denmark; 3 Department of Orthopaedics, Aarhus University Hospital, Palle Juul-Jensens Boulevard 99, 8200 Aarhus, Denmark

**Keywords:** Medical research, Bone

## Abstract

Recent studies have shown that distal radius fractures (DRFs) in elderly patients can be treated nonoperatively with good functional results after 1 year. However, scientific evidence regarding longer follow-up to assess posttraumatic arthritis (PA), complications, and functional outcomes is scarce. This prospective case series aimed to evaluate these outcomes in a cohort of patients ≥ 65-year-old with nonoperatively treated DRFs after a minimum of 3 years. The primary outcome was PA. Secondary outcomes were complications, Quick Disabilities of the Arm, Shoulder and Hand Outcome Measure (QuickDASH), Patient-Rated Wrist/Hand Evaluation (PRWHE), pain, range of motion and grip strength. The full data of 32 patients with a mean follow-up of 3.3 years were available: 10/32 patients had radiological signs of PA, but only 2 of these patients reported pain. A total of 11/32 fractures healed in malunion (> 10° dorsal angulation). There was no significant difference in QuickDASH or PRWHE from 1 year to the latest follow-up after more than 3 years. This study thus adds to the literature stating that radiological signs, including PA and malunion, do not necessarily result in symptoms. Moreover, it underpins that nonoperative treatment of these patients results in good functional outcomes after 1 and 3 years.

## Introduction

Distal radius fractures (DRFs) account for 18% of all fractures in elderly individuals ≥ 65 years of age^1,2^. The estimated lifetime risk for having a DRF is 15% for females and 2% for males^3^. The incidence rate is approximately 200 per 100,000 person-years^4,5^. Low-energy DRFs are associated with osteoporosis, and there is an age-related incidence rate that increases almost threefold from the age of 60 to 99 among women^1,4,6,7^.

The standard treatment in Denmark for displaced DRFs is surgery with open reduction and internal fixation (ORIF) to obtain anatomic reduction, but that treatment has been debated^8–10^. Studies have shown that elderly patients might not benefit from surgery, despite displacement of the fracture and indication for surgery based on Danish National Clinical Guidelines (NCGs)^11^.

Recent randomized controlled trials (RCTs) and meta-analyses on treatment of DRFs evaluating pain, patient-related outcome measures (PROMs), range of motion (ROM) or complications had a follow-up of 12 months, and PROM data after a longer follow-up are lacking^12–14^.

Posttraumatic arthritis (PA) may occur after fractures and even more so after intraarticular fractures. In 1986, Knirk and Jupiter published data on PA with an estimated prevalence of 65% after a mean follow-up of 6 years^15^. However, these fractures were high-energy fractures in a relatively young population of 25–52-year-olds. Knirk and Jupiter also described radiological predictors for PA as signs of articular incongruity, articular step-off in millimeters and lack of anatomical repositioning, which have informed clinical guidelines aiming to minimize these changes by either closed reduction or operative treatment. Among patients treated with ORIF, PA has been shown to be associated with pain and limited ROM, especially flexion and radial deviation^15,16^. Notably, no previous studies have described PA or predictors of PA among elderly patients with low-energy DRFs.

The aim of this study was to assess PA after a minimum observation period of 3 years among elderly patients after nonoperative treatment of low-energy DRFs. The secondary aims were to estimate complications and functional outcomes; the former was thought to increase and the latter decrease due to PA impairing activities of daily living.

## Materials and methods

### Setting

Health care in Denmark is fully tax funded and allows free and equal access for the country’s 5.7 million inhabitants. The Danish NCGs for DRFs advocate operative treatment if one of the following radiological parameters is met after closed reduction: (1) > 10° dorsal tilt of the radius perpendicular to the longitudinal axis of the radius; (2) > 2-mm articular step-off; (3) > 2-mm ulnar variance; (4) incongruence of the distal radioulnar joint; or (5) substantial dorsal comminution indicating gross instability.

### Design

This was a prospective case series of 50 patients with a minimum 3-year follow-up. The complication rate and functional outcomes of this cohort after 1 year have previously been published^17^.

All patients older than 65 years of age with a low-energy DRF who did not fulfill the radiologic criteria for surgical treatment according to the NCGs admitted to Randers Regional Hospital between November 2018 and July 2019 were screened for eligibility. If necessary, according to the NCGs, closed reduction/manipulation was performed under local anesthesia, i.e., a hematoma block using 5–10 mL of 20 mg/mL lidocaine without epinephrine injected at the fracture site. After 5–15 min the treating physician had a maximum of two attempts of closed reduction under fluoroscopic guidance. The patient was approved as eligible for this study by a member of the investigation group.

Exclusion criteria were secondary displacement of the fracture at two weeks follow-up, high-energy fractures, open fractures, concomitant injuries, e.g., multiple fractures, not being capable of giving written consent, and previous DRF or forearm fracture on the same arm.

### Recruitment and intervention

A total of 62 patients were included in the original study; 12 were excluded mainly due to fracture dislocation and operation after the first two weeks, leaving 50 patients in the study cohort. During the follow-up period from 6 to 12 months, another 2 patients were excluded due to death, leaving 48 for the follow-up visit at 12 months.

The present study is a follow-up of this cohort minimum three years after inclusion in the original study. The patients were contacted by telephone and invited to participate in the present study including a follow-up in the outpatient clinic.

#### Loss to follow-up from 1 to 3 years

Of 48 patients with 1-year follow-up of original study, 5 had died, 3 could not be reached, and 5 withdrew their consent to participate in the 3-year follow-up. Thirty-five patients gave consent on the telephone; however, 3 patients did not show up and further attempts to reach the patients by telephone were unsuccessful.

**Figure 1:**
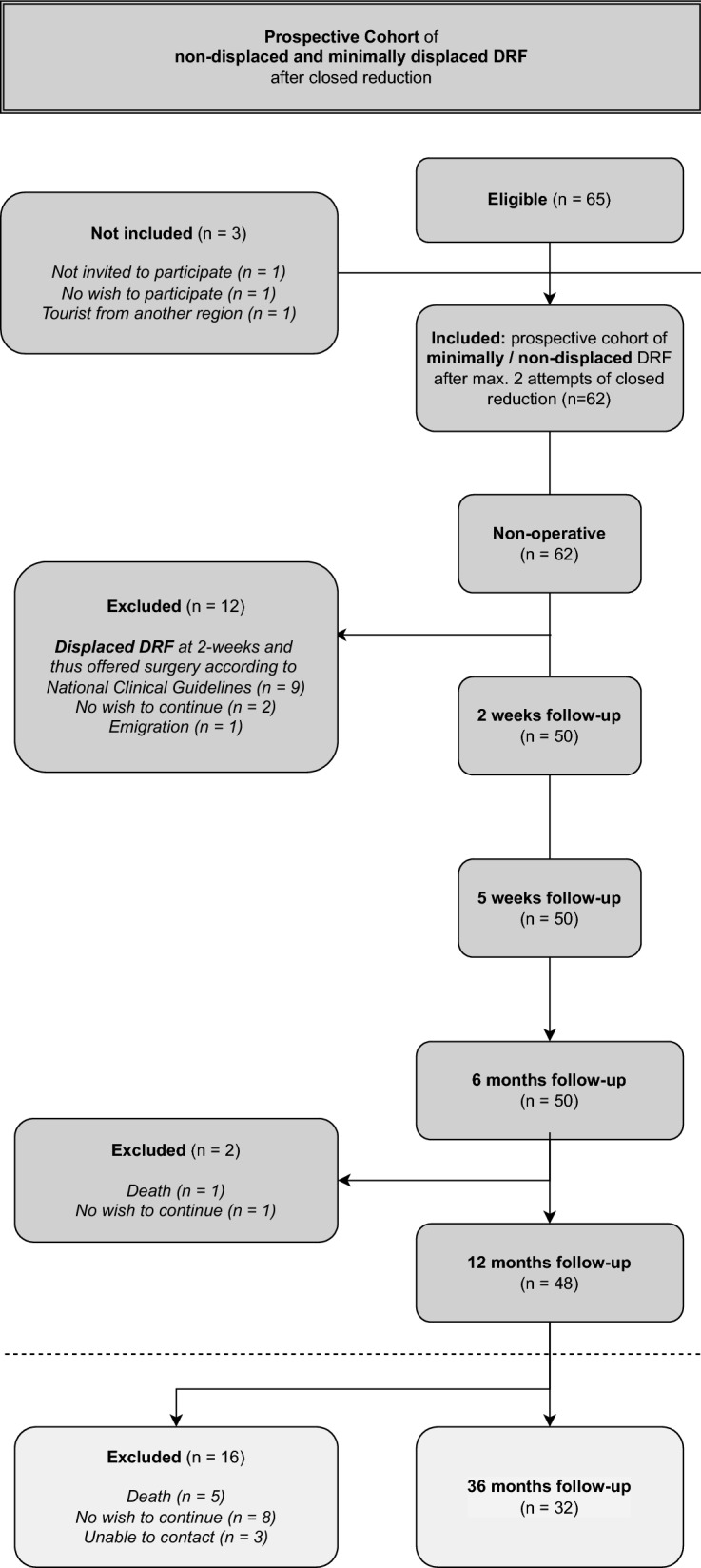
CONSORT flowchart.

The full data of the remaining 32 patients with at least 3 years of follow-up were available (Fig. 1). Demographic information is available in Table 1.

**Table 1:** Basic demographics.

Median age (min–max)	73 (66–86)
Female/Male	26/6
ASA: 1/2/3/4/5/6	11/18/3/0/0/0
Injured side: Right/Left	12/20
Working status: Retired/working	30/2
Smoker: Yes/No	4/28
Alcohol above limits: Yes/No	3/29

American Society of Anesthesiologists’ Physical Status Classification (ASA).

## Outcome measures

The following PROMs were evaluated: Quick Disabilities of the Arm, Shoulder and Hand Outcome Measure (QuickDASH) score, Patient-Rated Wrist/Hand Evaluation (PRWHE) score, and pain assessed on a numeric rating scale (NRS) of 0–10. ROM was assessed blinded as described in the published protocol. The complications form was filled out by a physician and nurse together with the patient. Standardized radiographs (anterior–posterior and lateral projections) of the distal radius were acquired.

This study complied with the Declaration of Helsinki and was approved by the Danish Scientific Ethical Committee as an extension of the study protocol (number: 1-10-72–420-17/79290, approved on 7th June 2021)^18^. Accordingly, all patients gave their informed consent.

### Primary outcomes

PA was the primary outcome. Standardized radiographs in two projections (anterior–posterior and lateral) were assessed by two consultants, one trauma surgeon and one hand surgeon. PA was rated according to Knirk and Jupiter^15^, where 0 equaled “none”, 1 equaled “slight joint space narrowing”, 2 equaled “marked joint space narrowing”, and 3 equaled “bone on bone contact”^6^.

The change in PA was assessed over time, i.e., radiographs taken 5 weeks after the fracture and the latest radiographs with a minimum of 3 years of follow-up.

### Secondary outcomes

On the radiographs, dorsal tilt and radial length were measured as previously described. The fractures were classified according to the AO Foundation/Orthopaedic Trauma Association (AO/OTA) fracture classification.

Complications were assessed at the 3-year follow-up in the outpatient clinic. Complications were defined as flexor or extensor tendon rupture or irritations, vascular compromise or sensory disturbance, including carpal tunnel syndrome and chronic regional pain syndrome, any associated operation during follow-up, and infection (superficial or deep). All subjective and objective complications were recorded. Medical journals were also assessed to obtain potentially missed complications.

ROM, i.e., wrist flexion, extension, pronation, supination, radial deviation, and ulnar deviation, was measured with a goniometer. The ROM of the contralateral wrist served as a reference.

Grip strength was measured using an electronic hand dynamometer (EH101 CAMRY). Grip strength was given as the mean of three measurements on each side. The minimal clinically important difference (MCID) of grip strength was set to 6.5 kg^19–21^.

Pain related to the fracture was reported on an NRS from 0 to 10. 0 was equal to “no pain”, and 10 was equal to “the worst pain one could imagine”. Pain was defined as the pain at the time of the examination.

The validated version of the Danish QuickDASH was used to assess the level of functionality and was self-reported by the patient. The MCID was a 16-point difference in QuickDASH^22–24^.

The validated Danish version of the PRWHE was employed as a self-reported assessment of five items on pain, 10 items on function and two optional items on appearance of the hand^23,25,26^. The MCID for the PRWHE was set to 10 points^25^.

### Statistical analysis

The mean and 95% confidence interval (95% CI) are given. Fisher’s exact test was used to compare the primary outcome after 5 weeks versus 3 years. The secondary outcome measure, complication rates, was also assessed with Fisher’s exact test. One-way repeated measures ANOVA including Sidak’s multiple comparison test was employed for the repeated QuickDASH and PRWHE values of the 32 patients with a complete follow-up. The statistical significance level was set to 0.05. GraphPad Prism version 9.5.0 for macOS was used for statistical analysis.

## Results

### Primary outcome

**Figure 2:**
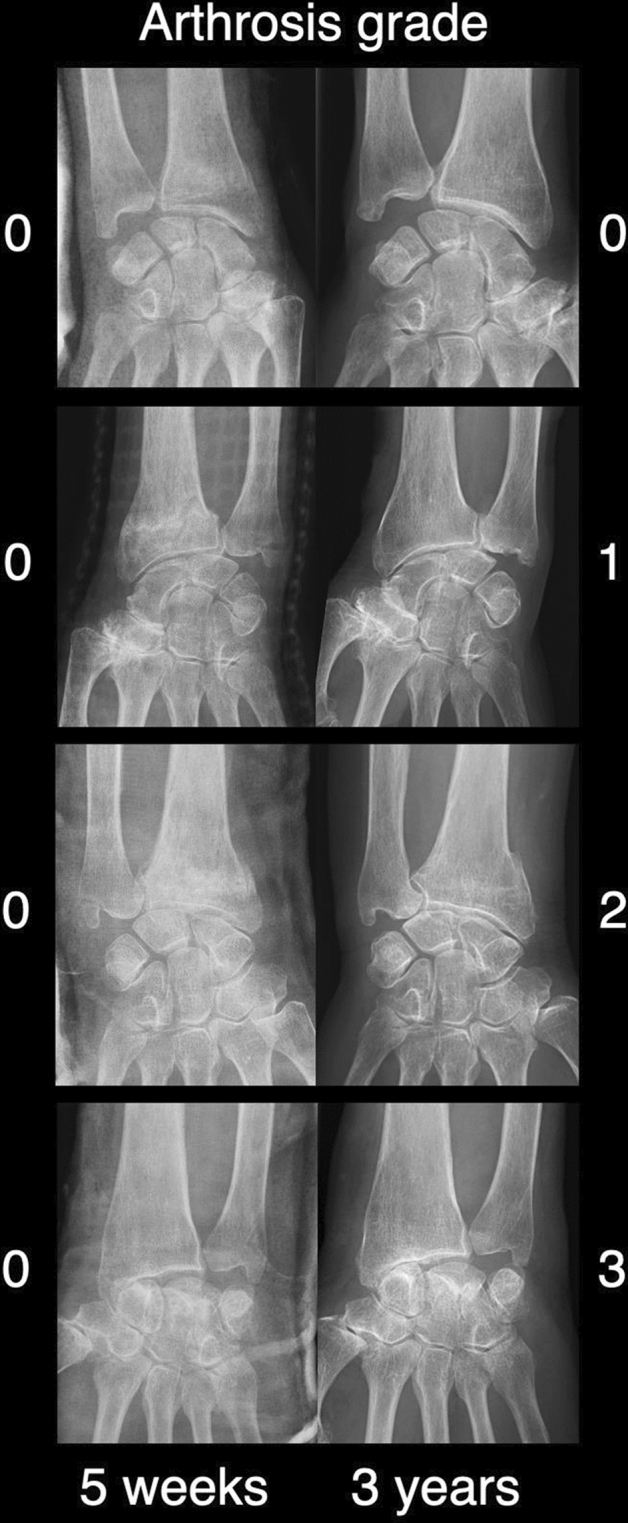
Examples of assessed anteroposterior radiographs with posttraumatic arthritis (PA) grades 0, 1, 2, and 3 after 5 weeks and a mean of 3.3 years after the injury.

In total, 10 out of 32 wrists had signs of PA after a mean follow-up time of 3.3 years (95% CI: 3.1–3.4; min. 3.0; max. 4.1). Arthritis was not evident in any of the 32 wrists 5 weeks post-injury (Fig. 2). At the latest follow-up, 7 wrists were rated as PA grade 1, 2 as PA grade 2, and 1 as PA grade 3. This change was statistically significant, i.e., 0/32 patients after 5 weeks and 10/32 patients after 3 years had radiological signs of wrist arthritis (Fisher’s exact test, *p* < 0.001). For details and the association between PA and pain please refer to Table 2.

**Table 2:** Posttraumatic arthritis (PA) and its association with pain.

PA grade	N = 32 (5 weeks)	N = 32 (3 years)	Pain (3 years)
PA 0	32	22	3 out of 22 patients: NRS 4;3;1
PA 1	0	7	1 out of 7 patients: NRS 1
PA 2	0	2	1 out of 2 patients: NRS 2
PA 3	0	1	0 out of 1 patients: NRS 0

Number of patients with posttraumatic arthritis (PA) and number of patients reporting pain after 3 years given on a numeric rating scale (NRS) 0–10.

### Secondary outcomes

The radiological evaluation after 3 years revealed a median dorsal angulation of 5 degrees (range: 15–24 degrees). Compared with the 5-week radiographs, the mean difference was -0.9 (95% CI: -5.6–3.8) degrees. The change from 5 weeks to 3 years was thus negatable for the vast majority of fractures. However, 11 out of 32 fractures healed with a dorsal angulation of ≥ 10 degrees. Five of these had radiological signs of PA on the latest radiographs. The 32 fractures were rated according to the AO/OTA classification: 12 were rated as A2, 11 were rated as A3, 1 was rated as B1, 4 were rated as B2 and 4 were rated as B3. There were no C-type fractures. AO type A fractures accounted for 72% of the fractures, whereas type B fractures accounted for 28%.

Complications after 12 months of follow-up were reported by 3/48 (6%) patients, while 6/32 (19%) experienced a complication at the latest follow-up: 5 patients reported nonspecific sensory disturbances, and 1 patient complained about limited function due to decreased ROM. The observed difference in the complication rate between the 12-month and 36-month follow-ups was not statistically significant (Fisher’s exact test, *p* = 0.15). Moreover, there were no associated operations during the follow-up time.

**Figure 3:**
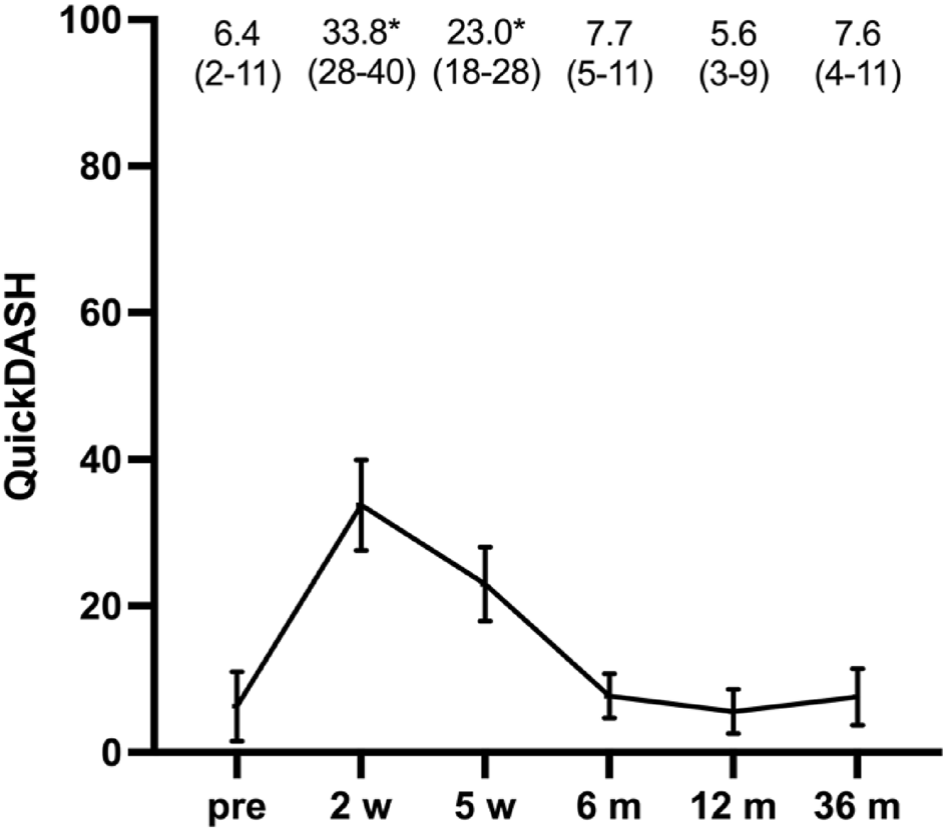
Mean QuickDASH score and 95% CI as error bars are given before the injury (pre) and after the injury at 2 and 5 weeks (w) and at 6, 12, and 36 months (m). *p < 0.05 compared with the preinjury state.

The mean QuickDASH values are given in Fig. 3 and did not significantly change from the 1-year follow-up to the 3-year follow-up. Moreover, one-way repeated measures ANOVA also showed that mean PRWHE values were comparable after 6, 12, and 36 months, i.e., 12.9 (95% CI 7.2–18.6), 9.1 (95% CI 3.8–14.5), and 9.0 (95% CI 4.3–13.6), respectively (*p* = 0.25).

## Discussion

In the present study, 10/32 patients had radiological signs of PA after 3 years of observation of nonoperatively treated low-energy DRFs among elderly patients. Notably, none of the patients had PA on the radiographs taken 5 weeks after the injury. Based on the PA classification by Knirk and Jupiter, one can expect that DRFs, especially intraarticular fractures and high-energy fractures, lead to PA in the majority of these fractured wrists^15^.

In the present study, 7 of the patients developed grade 1 arthritis, 2 of whom had an extraarticular fracture (type A) and 5 of whom had partially intraarticular fractures (type B) according to the AO/OTA fracture classification. Grade 2 arthritis was found in A2 and A3 fractures, and grade 3 arthritis was found in a B2 fracture. In total, 6 out of 10 cases of PA were observed in partially intraarticular fractures, while there were no C fractures in the cohort.

In agreement with our observation, Lameijer et al. described that intraarticular fractures with articular incongruence and older age were predictors of PA^27^. However, the systematic review found no correlation between AO/OTA classification of the fracture and development of PA and no prediction of PA or dorsal angulation, radial length, ulnar variance or radial inclination. Due to the unexpectedly low rate of PA and limited number of patients, we did not attempt to correlate PA grade and type of fracture.

The clinical impact of PA after low-energy DRFs in elderly people may be limited. In our study, only 2/10 patients with radiological PA reported pain. Van Leerdam et al.^28^ also described that type A and B fractures with a mean follow-up of almost 4 years had better PROMs when treated nonoperatively compared to operation among elderly patients. Our study and the study by Marchewka et al.^29^ align with this hypothesis, as approximately one-third of the wrists healed in malunion but with a good functional outcome and almost no pain. However, the role of malunion is a matter of debate, as other authors have found an association between radiological parameters and functional outcome^30–32^. We also found no statistically significant deterioration in functional outcome, i.e., QuickDASH and PRWHE scores, after 1 year compared with 3 years.

To our knowledge, there are only a few studies with a follow-up of more than 3 years for DRFs treated with or without surgery in elderly patients. Previous publications have had only a 1-year follow-up and showed good results in terms of PROMs and few complications^12,13,17^. It may be argued that the follow-up period should be even longer than 3 years before PA becomes symptomatic. However, a study from 2008 among younger patients supports the theory that malunion and radiological signs of PA do not necessarily result in symptoms even after more than 30 years^33^.

We noticed a nonsignificant increase in the complication rate from 3/48 (6%) patients after 12 months to 6/32 (19%) patients after 36 months. All complications were minor, consisting mostly of sensory disturbances. None of these applied to specific nerves, and none of the patients with sensory disturbances required secondary surgery. In comparison, we have previously reported a complication rate of 15% in operatively treated DRFs with a 3.2-year follow-up. However, that study was retrospective, and almost 10% of DRFs required reoperation due to major complications^4^. The complication rate in the present study is comparable to that in earlier published studies with a shorter follow-up^14,34,35^.

Limitations of the present study include the size of the patient cohort as we saw loss to follow-up of 18 out of 50 patients over the 3-year follow-up period. However, this study was an extension of a well-designed study with the primary aim of assessing complications after 12 months. Second, arthritis was graded by 5-week radiographs while the wrist was still in a cast, i.e., standardized radiographs taken to assess the healing of the fracture before cast removal. Evaluating arthritis with these radiographs may have obscured subtle signs of arthritis. Another limitation could be the unawareness of the patients’ comorbidities, such as rheumatoid arthritis or pain and disability from basilar thumb arthritis. Assessment of the contralateral wrist by standardized PROMs and radiographs may partly have overcome this limitation. However, PROMs such as QuickDASH score are not side specific but assess the patient’s ability to perform activities of daily living regardless of whether these activities are performed with the healthy or injured side^36^.

A strength of the present study is its follow-up time of 3 years, which is long compared with the majority of other DRF studies. Moreover, the study design was prospective and thus accounted for even minor and rather nonspecific changes in, for example, complications such as sensory disturbances. Moreover, the loss to follow-up was low compared to the literature. From the 1-year follow-up until the final follow-up, only 16 patients dropped out, and 7 died. In comparison, the loss to follow-up over a 3-year period was 65% in a recent study from 2022^28^.

## Conclusion

PA was observed in 10/32 (31%) wrists after low-energy, nonoperatively treated DRFs in patients older than 65 years of age after a minimum follow-up of 3 years. None of the patients had arthritis based on the 5-week postinjury radiographs. Notably, only 2 of the 10 patients with PA complained about any, i.e., mild pain and their good functional outcomes (QuickDASH and PRWHE scores) after 1 year did not deteriorate over time. Despite the small population, this study thus adds to the literature stating that radiological signs, including PA and malunion, do not necessarily result in symptoms. Moreover, it underpins that nonoperative treatment of these patients results in good functional outcomes after 1 and 3 years.

## Data Availability

Anonymized data may be requested from the corresponding authors R.T. and J.D.R.
